# Valedictory Address to the Graduates of the Medical Department of Pennsylvania College

**Published:** 1840-03-28

**Authors:** 


### BIBLIOGRAPHICAL NOTICE.

VALEDICTORY ADDRESS *to the Graduates of the Medical Department of Pennsylvania College.* By WILLIAM RUSH, M. D., Professor, &c.

ONE of the most spirited and agreeable addresses that we have for a long time had the pleasure to peruse, is the valedictory of Professor Rush, recently published by the class of the medical department of Pennsylvania college. A somewhat hackneyed topic—the prospects and difficulties of a physician's career—is treated by the professor with much originality and liveliness of thought and style. We select a few extracts:—

*The turn of mind proper for a Physician.*—"It may be asserted, that the vocations of men are generally induced by early and corresponding inclination for them; and though the exception is proved in the accidents which change their pursuits, and influence their destinies, biography frequently classes, as cause and effect, an early turn or bent of mind, and the subsequent strength of its grasp. Eminence, in every department of science, has traced its first risings to such early mental predilections, and it is fortunate for the young man, destined for the Medical Profession, if he brings to its elementary studies, powers of investigation, previously exercised upon *things*, without new and *formidable names.* Words and terms, are to him, in his new enterprise, as different strata of earth to the miner. He learns them only to know their want of use, or their frequent hindrance to his progress. But knowledge gained through the avenues of his senses—facts collected from Nature's undoubted proofs, these are the student's gold region, these, the riches which reward his toil. I need not loiter here upon the path leading to his medical degree, in attributing to him a sixth sense, if I may so speak, in the power of prompt investigation through his other five: I allot to him the best means of simplifying knowledge, and rendering its acquisition practical.

There is a resemblance in the minds of those eminent in the Medical Profession, to those distinguished in Natural Science, and the Mechanic Arts—but, the votaries of the two latter, have in their pursuit, greater facilities to their advancement, they are less obstructed by the clogs of unmeaning terminology, or the encumbrances of abstract theories. It is through experiment by the hand, the eye, by all the senses, through tests, generally the result of patient purpose; and sometimes, as if to crown with fortune such wise methods of investigation,—of very accident—that science has been developed in her simplicity, and her usefulness. These tests have been to scientific improvemnnt, the giant strides with which she has marched. The student of medicine, aware of this, by practical observation, triumphs over his relative disadvantages, and adheres to it as the best assurance of advancing truth. His authority for it, is the reason of the rule, his examples of its achievements, whether in Medicine or Philosophy, are in the illustrious names of an Hippocrates or a Galen; a Bacon or a Newton."

*The Physician's difficulties in youth.*—"I name among his early discouragements—the prevalent opinion that a Physician must be of a certain age, not in attainments, or power to use them, but in name—and number of years before he shall be entitled to confidence, employment, or reward. He may have passed his medical youth and manhood in hospitals, and among their myriads of patients;—with heroic zeal he may have walked amidst the modern pestilence of Asia, and of Europe, learning, through perilous experience, more than a life might teach; still I will carry the familiar conjecture so far as almost to believe, he will return to his native land and ask for confidence in vain,—unless an Indian clime has furrowed his cheek,—or the frosts of Russia have touched the hairs of his head. Why is not this distrust of youth exhibited in other vocations? We ask not the age of those, to whom is entrusted the care of our immortal souls; nor of those whose duty it is to protect our rights—and even to save us from an ignominious death!—their youth is a warrant for their zeal and activity."

*The Physician's reward in age.*—"I have said that the usefulness of the practitioner of medicine passes not away in his old age. Time, then, proves to him an equitable paymaster. He is to his latest hour requited for confidence withheld in youth, and, if I may so speak, with interest too, for its early deprivation,—age dims not his vision of disease, nor of the means of its cure; and when his tottering limbs bear him to the door of his patient—it is a sign true as triumphant to the venerable physician, that a life of exceeding value is in jeopardy within. The ripeness of his years, is the fulness of his honor; and in that unalloyed homage, received by illustrious men in their latter days, he enjoys what has been happily called "the best foretaste of Immortality." As surely as in his path of life, there are many "ways of pleasantness," so in its end, there is peace. Many, many, mourn his loss, and his memory is a monument reared by death, to his vanquished but intrepid enemy."

